# Exploring the Role of Health Expenditure and Maternal Mortality in South Asian Countries: An Approach towards Shaping Better Health Policy

**DOI:** 10.3390/ijerph182111514

**Published:** 2021-11-02

**Authors:** Noshaba Aziz, Jun He, Tanwne Sarker, Hongguang Sui

**Affiliations:** 1 College of Economics and Management, Nanjing Agricultural University, Nanjing 210095, China; noshabaaziz@yahoo.com; 2 School of Economics and Finance, Xi’an Jiaotong University, Xi’an 710049, China; sarker@stu.xjtu.edu.cn; 3 School of Economics, Shandong University, Jinan 250100, China; hongguang.sui@sdu.edu.cn

**Keywords:** health expenditure, maternal mortality, sanitation, clean technologies, South Asian countries

## Abstract

Accomplishing unremitting favorable health outcomes, especially reducing maternal mortality, remains a challenge for South Asian countries. This study explores the relationship between health expenditure and maternal mortality by using data set consisting of 18 years from 2000 to 2017. Fully modified ordinary least squares (FMOLS) and dynamic ordinary least squares (DOLS) models were employed for the empirical analysis. The outcomes revealed that a 1% rise in health expenditure increased the maternal mortality rate by 1.95% in the case of FMOLS estimator and 0.16% in the case of DOLS estimator. This reflects that the prevailing health care system is not adequate for reducing maternal mortality. Moreover, the meager system and the priorities established by an elitist system in which the powerless and poor are not considered may also lead to worsen the situation. In addition, the study also added population, economic growth, sanitation, and clean fuel technology in the empirical model. The findings revealed that population growth has a significant long-term effect on maternal mortality—an increase of 40% in the case of FMOLS and 10% in the case of DOLS—and infers that an increase in population growth has also dampened efforts towards reducing maternal mortality in the South Asian panel. Further, the results in the case of economic growth, sanitation, and clean fuel technologies showed significant long-term negative effects on maternal mortality by 94%, 7.2%, and 11%, respectively, in the case of the FMOLS estimator, and 18%, 1.9%, and 5%, respectively, in the case of the DOLS estimator. The findings imply that GDP and access to sanitation and clean fuel technologies are more nuanced in declining maternal mortality. In conclusion, the verdict shows that policymakers should formulate policies considering the fundamental South Asian aspects warranted to reduce maternal mortality.

## 1. Introduction

Human capital is an imperative factor for attaining the desired economic growth and development of any country [1]. Under the concept of the neoclassical growth model, human capital, particularly good human health, markedly influences a country’s per capita income in the long run [2]. The health status of any given country depends upon maternal and infant mortality rates, which play a crucial role in evaluating the country’s population health, quality of care, socioeconomic status, and poverty [3]. Moreover, maternal health is likely to bring economic benefits for the whole family and society. But recently, mothers’ poor health, especially maternal deaths, have remained an unavoidable phenomenon that occurs at relatively high rates in many developing countries despite a steep reduction in maternal mortality worldwide since 1990 [4,5,6]. The maternal mortality ratio numerically shows the maternal deaths during a given time period per 100,000 live births during the same time period. It illustrates the maternal death risks relative to the number of live births and basically captures the death risk in a single pregnancy or a single live birth. According to the World Health Organization (WHO), around 810 women die during pregnancy every year, and about 94% of them belong to developing countries [7].

Globally, the maternal mortality ratio has declined from 342 to 311 (by nearly 38%) from 2000 to 2017 [7]. However, South Asian countries and sub-Saharan African countries face disproportionately higher maternal mortality ratios than other regions of the world, making up around 85% of all maternal-related deaths. The third Sustainable Development Goal (SDG 3) of reducing the deaths to 70 per 100,000 live births and ensuring that all human beings, at all ages, achieve healthy lives by 2030, has intensified their struggles to promote the health sector [8]. In this regard, a good health care system is focused, and has been is shown that the health care system comprises various sectors, of which the most important is health financing [3]. Evidence has confirmed that financing in the health sector considerably supports the economy [9]. This implies that spending in the health care system improves peoples’ health and generates employment opportunities, increases social and political stability, and ultimately leads to the growth and development of the whole economy [10,11,12]. 

Despite significant investments in the health sector, expenditure on health in governments’ financial plans in developing regions, including South Asian countries, is still under-represented due to resource scarcity [13]. Even if South Asian countries develop and progress at the same pace, the global SDG target of decreasing maternal deaths to 70 per 100,000 live births by 2030 is hard to accomplish. Over the past few decades, keeping in view the importance of the health of the people and its role in the national economy, scholars and researchers have devoted their attention to evaluating the link between health expenditure and health outcomes. In this vein, many studies have been conducted before (see, for example, [14,15,16,17,18,19,20,21]). Some studies showed that health expenditure leads to better health outcomes [22], while others found no such relationship [23,24]. The link between health expenditure with health outcomes is uncertain, as health expenditure and health care patterns alter considerably; therefore, the debate over health care expenditure and health outcomes remains questionable [25], which warrants more investigation. Moreover, the rise in health expenditure in developing countries has demonstrated that maternal mortality rates are still higher, which has prompted authors to identify the core cause of maternal mortality, so health expenditure is appropriately directed and utilized. 

Based on the above discussion, the current study was designed to explore the following research question: “Does health expenditure contribute to decreased maternal mortality in South Asian countries?” Exploring this question will enable policymakers to prepare strategies accordingly to accomplish the sustainable development goal of reducing maternal mortality, as health expenditure is regarded as the clearest policy variable [26]. Investigating health care outcomes in South Asian countries is remarkably important, not only due to the relationship of health with economic growth but also poor health leads to disparity between actual income and real opportunities, as it decreases the degree to which a given income level can be transformed into the ability to live a life of an acceptable quality [27]. Perkins, Radelet, Lindauer, and Block [28], and Booth and Cammack [29] also pointed out that the health sector is among the most pervasive markets that may lead to failure because of the problems of principal agents, negative externalities, collective actions, and information failures, which require government interventions. Moreover, most preceding studies ignored other macro-level factors of maternal mortality, such as population growth, economic growth, sanitation, and access to clean fuel technologies. Thus, the current study may help to unpack the association of health expenditure with maternal mortality by incorporating these factors for the South Asian region. 

As discussed above, many previous studies have explored the relationships between health outcomes (mortality) by using different datasets and have used different research methods and found diverse findings, particularly for the maternal mortality ratio [24,30,31,32,33,34]. However, a recent study pointed out that an investigation of this kind must account for “unobserved heterogeneity” [34]. Even so, many studies have ignored the heterogeneity and even cross-sectional dependency within the panel data. Therefore, to fix the cross-sectional dependency and heterogeneity issues in the panel data and add a contribution to the health economics literature, the present study applied fully modified ordinary least squares (FMOLS) and dynamic ordinary least squares (DOLS). The recent study of Aziz et al. [35] also used this phenomenon to assess the determinants of undernourishment in a South Asian panel. As per the authors’ knowledge, this is the first study attempting to scrutinize the long-run effects of health expenditure on maternal mortality in a South Asian panel via this empirical approach.

## 2. Literature Review

This section is an attempt to review the previous efforts of scholars and researchers on the subject matter of the current study. This section concisely reviews the studies available in the literature. 

According to previous studies, the health expenditure level in a nation is one of the key measures for determining the health investment level; hence, it is acknowledged as an imperative input factor, like diet and exercising, for enhancing health. An extensive body of literature has scrutinized the association between health expenditure and health outcomes across countries. Despite these efforts, the pivotal link between health expenditure and health outcomes is still not clear. It is generally presumed that increasing health expenditure will inevitably improve health outcomes. In this regard, Aldogan, Austill, and Kocakülâh [36] observed prominent health outcomes such as infant, under-five, and maternal mortality rates due to government spending in the MENA region from 1990 to 2010, by using pooled ordinary least regression, random effects, and Hausman–Taylor instrumental variable models. Betrán et al. [30] argued that with the exception of developed countries, variability of national maternal mortality estimates is large even within sub-regions. In another study, Anyanwu and Erhijakpor [9] used data from 47 African countries between 1999 and 2004, and statistically revealed that health expenditure significantly and negatively influenced the infant and under-five mortality rates. In developing countries, Bokhari, Gai, and Gottret [37], using the instrumental variable approach, implied that economic growth and government expenditure on health are important factors for boosting public health. Nketiah-Amponsah [38] studied 46 sub-Saharan African countries over the period 2000–2015 and unveiled that a 1% increase in health expenditure per capita reduced under-five mortality by 0.5% and maternal mortality by 0.35%, while improving life expectancy by 0.06%. Likewise, Arthur and Oaikhenan [39] also indicated that health expenditure meaningfully boosted the expectancy of life and lessened maternal mortality and under-five mortality. 

On the other side, several other studies found no causal association between expenditure on health and health outcomes [24,40]. Ashiabi, Nketiah-Amponsah, and Senadza [41], in another study, investigated the impact of both public and private health expenditure on maternal and child health for 40 sub-Saharan African countries for the years 2000–2010. The study used a fixed effect model and indicated that there was no significant effect of health expenditure on maternal mortality. Bradley, Elkins, Herrin, and Elbel [42] used a pooled cross-sectional analysis of OECD countries from the year 1995 to 2005. The main outcomes were life expectancy at birth, infant mortality, low birth weight, maternal mortality and potential years of lost life. The findings revealed that health service expenditure adjusted for GDP and two health outcomes were significantly associated, while in the case of social services expenditure adjusted for GDP; only three of five indicators were found to be significant. Several other studies have also shown contrary findings in low- and high-income countries. For example, Self and Grabowski [43] revealed that health expenditure significantly influenced health only in low- to middle-income countries. The countries belongs to different income groups have differences in health expenditures as well which corresponds to different health outcomes. Likewise, Rana, Alam, and Gow [34] indicated that the association of health expenditure with health outcomes was resilient only for countries with a low income. Lower-income countries are more susceptible to poor health due to negative shocks to health expenditure. Gupta, Verhoeven, and Tiongson [44], by using demographic health surveys from 44 countries, established that the poor experience worse health than the non-poor, and their empirical findings stated that public spending alone is not enough to improve health status. Gupta [45] argued that public spending enhances health and education in developing and emerging economies. Ullah et al. [46] findings confirm that public healthcare spending significantly impacts health outcomes in Pakistan both in the short-run and long-run. Using a fixed effect model, Nixon and Ulmann [19], in a panel of 15 European Union members over the period 1980–1995, showed that health care expenditure and infant mortality rates were strongly associated, while the results for life expectancy were found to be marginally associated. The findings indicated that factors such as lifestyle, diet, and the environment also influence health outcomes, as these factors vary significantly between low-income and high countries. Therefore, the relationship between health expenditure and health outcomes at different income levels was found to be inconclusive. In another study of Nigeria, Yaqub, Ojapinwa, and Yussuff [47] stated that public expenditure was significant for enhancing health unless corruption in the country was controlled. By using both ordinary least squares and two-stage least squares, the study proposed that lowering infant mortality and under-five mortality rate and raising the life expectancy in Nigeria would only be possible if corruption in the country reduced considerably.

Based on the literature review mentioned above, it is apparent that the results on heath expenditure and health outcome are inconclusive and there has been variance in the use of specific econometric methods. To explore the phenomenon empirically by using advanced econometric techniques, the current study utilized the data of South Asian countries. Finally, by using a more robust empirical strategy, the current study offers new empirical evidence concerning health expenditures and it impact on maternal mortality in the South Asian panel.

## 3. Materials and Methods

### 3.1. Data Sources

Based on the availability, the data set of eight South Asian countries including Pakistan, Bangladesh, Bhutan, Nepal, Sri Lanka, India, Maldives and Afghanistan was collected. The maternal mortality ratio (MMR) was used as an outcome variable; this numerically shows the women died during pregnancy or pregnancy-related causes or within 42 days of pregnancy termination per 100,000 live births, while the predictor variables included total health expenditure (HE), population (POP), economic growth (LNGDP), basic sanitation (BS), and access to clean technologies (CT). Health expenditure was measured in current USD. Economic growth (LNGDP) was measured in constant 2010 USD and was used in natural logarithmic form for empirical analysis. The variable population (POP) was measured as the annual percentage growth in the population. The other variables, basic sanitation (BS) and clean technologies (CT), measured the proportion of the population using at least basic sanitation services and clean fuel technologies for cooking. 

**Figure 1:**
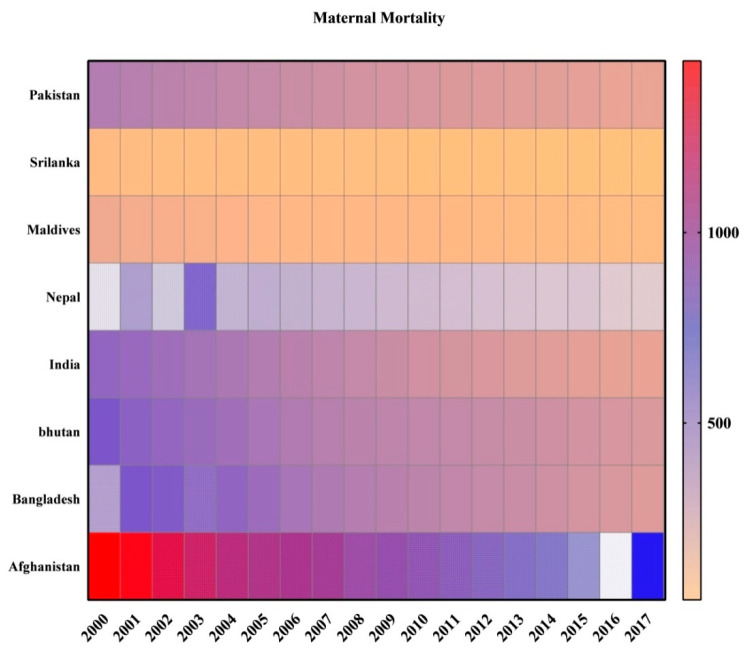
Trends of Maternal Mortality in the South Asian Panel.

The data for the desired variables used in the current study were sourced from the World Bank Development Indicators databank from 2000 to 2017. Furthermore, the quarterly data were used for applying the quadratic match sum method. Many previous studies have used this method [48]. This method is appropriate for altering low-frequency data into high-frequency data, as it allows adjustments for seasonal deviation by dropping end-to-end data deviation. The trends of maternal mortality and health expenditure in South Asian countries are portrayed below. The Figure 1 below reveals that though maternal mortality has slightly declined in all regions, the most apparent decline is seen for Afghanistan compared with other regions, and the least were seen in Sri Lanka and Maldives. 

### 3.2. Model Specification

It is a crucial and well recognized fact that a better understanding of various aspects in the economy leads to the formulation of effective economic policy, including the nexus among maternal mortality, health expenditure, economic growth, population, basic sanitation, and clean technologies. As the present study intended to explicate the long-run relationship of health expenditure effects on maternal mortality in South Asian regions while controlling for population, economic growth, basic sanitation, and access to clean technologies, the subsequent model was designed to analyze the relationship empirically:

*MMR_i,t_* = *α*_0_ + *β*_1_*MMR*_*i,t−*1_ + *β*_2_*HE_i,t_* + *β*_3_*LNGDP_i,t_* + *β*_4_*POP_i,t_* + *β*_5_*BS_i,t_* + *β*_6_*CT_i,t_* + *μ_i,t_* (1)

where MMR, HE, LNGDP, POP, BS, and CT signify the maternal mortality ratio, health expenditure, economic growth, population, basic sanitation, and clean fuel technology, respectively. In this model, the maternal mortality ratio represents the dependent variable; *βk* (*k* = 1, 2, 3, 4, 5) are the coefficients of the lag of maternal mortality ratio, health expenditure, economic growth, population, basic sanitation, and clean fuel technology; and *μ_i,t_* shows the error term. Our models remained confined to these variables to avoid over fitting the model and to allow our core variables of interest to show the association.

Determination of the long-run relationships among the study variables was undertaken via the following essential steps. Firstly, panel unit root tests were used to examine the stationarity properties of the variables. Testing stationarity is vital for both time and panel data because non-stationary data lead to spurious results [49]. Thus, Im, Pesaran, and Shin’s test [50] for unit roots was applied to test the stationarity [51,52,53]. However, it has been argued that such unit root tests for panel data may not be accurate due to the probability of cross-sectional dependency. Therefore, second-order generation tests such as cross-sectional IPS (CIPS) were also used, along with a cross-dependency test (CD), as these tests can be executed under the notion of cross-sectional dependency as well as heterogeneity [54], which was unnoticed by Im, Pesaran, and Shin [50]. Many earlier studies have used this method [55,56].

Once the stationarity had been checked, the next step was to test the variables’ cointegration. Cointegration expresses the association among the studied variables in the long run. The cointegration test of [57] for panel data involves two stages. In the first stage, it monitors short-run parameters and individual deterministic trends to control the heterogeneity. On the basis of the estimated residuals, statistics of different tests can be derived, i.e., pooled (within-dimension) data to test the common process, and grouped (between-dimensions) data to test individual processes. The four statistics included in a within-dimensions approach were panel v, panel ρ, panel PP, and panel ADF. In contrast, the three statistics included in the between-dimensions approach were group O, group PP, and group ADF. The cointegration test only indicated the long-run associations among the variables and did not indicate the direction of the variables used in the study. 

To indicate the direction, the study primarily used fixed effect and random effect models [58], but as the panel data suffered from cross-sectional dependency, FMOLS and DOLS were used, as in Kao and Chiang [59]. FMOLS and DOLS are appropriate for panel data. FMOLS is a non-parametric technique that deals with serial correlation, while DOLS is a parametric method that deals with endogeneity. 

## 4. Results and Discussion

### 4.1. Descriptive Statistics

The variables used in the current study and their descriptive statistics are presented in Table 1. The findings reveal that maternal mortality’s maximum and minimum values are around 1450 and 36, respectively, for the sample countries. The sampled countries’ health expenditure had a mean value of about 105, with a standard deviation of 184, a maximum of 946, and a minimum value of 8.36. The mean value of economic growth was USD 24.18; the mean value for population was nearly 1.83 and its standard deviation was about 1.067. Clean fuel technologies and basic sanitation had a mean value of 32.16 and 53.63, with a standard deviation of 20.24 and 23.83, respectively.

### 4.2. Unit Root Tests

Before performing the regression analysis, the variables’ stationarity was required to be checked. First, the variables’ stationarity properties were checked using the conventional unit root tests of Im, Pesaran, and Shin [50]. In Table 2, the findings of the unit root test with individual intercepts and individual intercepts with trend terms are illustrated. The findings reveal that basic sanitation and clean technologies have a unit root problem at the level but become stationary at first difference. The study then proceeded to inspect the cointegration among variables.

Before checking cointegration, the present study used the cross-sectional dependency. It has been argued that orthodox unit root tests do not offer robust outcomes in data series with cross-sectional dependence. Therefore, according to the cross-section dependency test results, it was apparent that there was cross-sectional dependence, as the results were found to be significant for all variables at the 1% significance level (see Table 3).For panel data with cross-sectional dependence, it is recommended to use a cross-sectional unit root test IPS (CIPS), rather than the conventional Im, Pesaran, and Shin unit root test. Therefore, the current study used the CIPS results, which assumed cross-section dependency within the panel data and revealed that all variables became stationary at first difference. 

### 4.3. Panel Cointegration Test

Once the stationarity features of all variables had been proven, panel data cointegration was valid to apply. To check the cointegration, the study used the panel cointegration test of Pedroni [57]. The results in Table 4 revealed that four tests out of seven were significant at the 1% level. Based on these test results, it is apparent that variables are co-integrated in the long run.

### 4.4. Panel Estimation Results

#### 4.4.1. Fixed Effect and Random Effect Results 

As a preliminary analysis, the conventional panel data models, i.e., fixed and random effects were applied. The results of the fixed effect model showed that health expenditure, economic growth, and population positively influenced maternal mortality in South Asian countries by 43%, 58%, and 71%, respectively, while in the case of random effects, the coefficients were similar: 42%, 44%, and 72%, respectively. The findings for health expenditure are unexpected and reveal that an increase in health expenditure increased maternal mortality in the South Asian panel. In contrast, access to sanitation and clean fuel technologies were proven to reduce maternal mortality. The random effect model also reported the same findings, with similar coefficients in the sample countries. Hausman’s [60] test was also executed, and indicated that the fixed effect model was more appropriate for the current study (see Table 5). However, as the panel data suffered from cross-sectional dependency and heterogeneity, as the countries are diverse and are not identical in their demographic aspects, health infrastructure, medical technology, and the prevalence of diseases, relying on fixed effects may give biased results. Therefore, the study proceeded towards using fully modified ordinary least squares and dynamic ordinary least squares. These methods are beneficial for overcoming heteroscedasticity and autocorrelation in the panel data [61,62]

#### 4.4.2. FMOLS and DOLS Results 

Table 6 reports the estimation results of FMOLS and DOLS and indicates that though the magnitude of the coefficients is diverse, the signs are almost similar. There is a consensus that increased health expenditure crucially reduces maternal mortality. However, in South Asian countries, the outcomes are quite surprising, showing that a 1% rise in health expenditure led to an upsurge of the mortality rate by 1.95% for FMOLS and 0.16% for the DOLS estimator. The results in the case of economic growth were, as expected, significant and vigorous, and negatively affected maternal mortality. The outcomes show that an increase in GDP by 1% negatively influenced maternal mortality by 94% in FMOLS. The results in the case of population were expected and revealed that a 1% increase in population growth increased maternal mortality by 40% in the case of FMOLS and 10% in the case of DOLS. In the context of basic sanitation and clean fuel technologies, the results were found to be negatively significant: 7% and 11% in the case of FMOLS and 1.9% and 5.6% in the case of the DOLS estimator. 

### 4.5. Discussion

This study contributes to the rising debate regarding maternal mortality and health expenditure. The data from eight South Asian countries over 2000–2017 is used for the FMOLS and DOLS estimation techniques. The study findings revealed that there is rise in the maternal mortality ratio in developing countries and it is less likely to obtain better health facilities. The results further imply that majority of females in developing countries belong to resource-poor settings, and even when the females reach a health center, they face innumerable hurdles such as inappropriate and inadequate care, and also other factors such as failures in the delivery of health services, a shortage of equipment or personnel, and—worst of all—faulty management. Despite increasing health expenditure in the sampled region, the unsatisfactory and meager quality of management and substandard health care services may dampen efforts to reduce maternal mortality. The result further suggests that though funds allocated for health care have increased in South Asian countries, the funds might be poorly distributed and mismanaged. Previous studies also found that a large share of the health budget is spent on pharmaceuticals, which reduces the share for other health programs [63]. Various other studies have shown contradictory findings regarding healthcare expenditure and health outcomes. For instance, Rana et al. [34], and Anand and Ravallion [64] revealed a positive link between health expenditure and the health sector’s performance. Nketiah-Amponsah [38] showed that an increase in expenditure on health of 1% resulted in reducing maternal mortality by 0.35% and under-five mortality by 0.5%, and improved life expectancy by 0.06%. 

Regarding infant mortality, the study revealed that total expenditure on health significantly reduced infant mortality in South Asian countries [65]. In contrast, Filmer, Pritchett, and Musgrove [24,40] found no association between these variables. Likewise, Zakir and Wunnava [66], and Nolte and Mckee [67] also found no association between health expenditure and health outcomes. Moreover, the health expenditure effects in the sampled region can additionally be attributed to the fact that there are culturally significant beliefs regarding prenatal care services. Most of the women in South Asian countries perceive prenatal care to be worse for their offspring in the womb. Thus, on the basis of empirical estimations, it was concluded that health and betterment in maternal health are not associated with increased health expenditure. Still, the value, cost, reliability, and acceptability of health facilities also matter. 

Similarly, the positive coefficient of population growth revealed the expected finding that an increase in population also leads to increased maternal mortality. South Asia comprises a considerable proportion of the global population and includes some of the peak child and maternal mortality rates internationally. The reason could be that when households have more family members, they may be less likely to provide adequate nutrition and healthcare services [68,69]. Such an alarming growth rate could lead to absolute scarcity of food, shelter, clothing, and, more importantly, health services. The exploitation of resources becomes worse due to population pressure and creates many social problems. Due to the large population size, most health services are required to be shared, and a large portion of the health expenditure is spent on curative medicines, which mostly reach the people of urban areas. Moreover, the majority of the population in developing countries belongs to resource-poor settings and face hurdles in obtaining government of health care services due to the great mass of inhabitants, so maternal death prevention entails fundamental changes not only through increased health expenditure but also proper resource allocation and delivery of health services. People from resource-poor settings will have to be fought for to achieve equity and social justice [70,71,72,73]. 

Setting health expenditure and population growth aside, the significance of economic growth, access to sanitation, and clean fuel technologies in decreasing mortality rate cannot be disregarded. This suggests that maternal mortality can be reduced with an increase in income. Moreover, dietary preferences and lifestyle can also be enhanced with an increased share of income [74,75]. Many prior studies have stated a similar verdict and revealed that economic growth increases the average income, which intensifies accessibility and consumption of goods and services, and eventually improves peoples’ health [76,77]. Increased income can help women seek experienced health experts and recognize the perils of not receiving satisfactory healthcare during pregnancy. The study of Pritchett and Summers [78] also showed that per capita GDP positively and significantly influenced health. Usually, in developing countries, people face financial constraints and cannot afford health expenses, so they prefer to spend on food commodities. Therefore, in this case, government spending can improve the health of poor people. Health is an essential human right, so economic growth can lessen maternal mortality in South Asian regions. Bloom, Canning, Kotschy, Prettner, and Schünemann [79] also showed that income positively affects health and improves life expectancy. The findings also showed that individual income plays a vital role in improving people’s health in South Asian countries. 

Further, several studies have shown a link between sanitation and improved health outcomes [80,81]. Many former researchers have considered access to water and sanitation for dropping the morbidity of diarrhea, and infants and under-five mortality [82,83,84]. The existing studies proposed that well-executed interventions in socioeconomic conditions, particularly in areas where primary settings are meager, can reduce maternal mortality. The results correspond well with those for sub-Saharan Africa found by Pickbourn and Ndikummana [85], where mortality rates and diarrhea morbidity were greater than in other regions. The individuals whose water and hygienic sanitation improved are among the lowest in the world. Thus, the findingsindicatethat investments in socioeconomic determinants such as sanitation and clean technologies are also likely to improve the maternal health status of the South Asian region.

## 5. Conclusions and Policy Recommendations

The mother’s health is crucial for a child’s cognitive growth and time spent caring for children. Given the rising maternal mortality rates in South Asian countries, reducing maternal mortality remains a noteworthy challenge for human development in South Asian countries. The current study unpacked the myth of increased health expenditure and reduced maternal mortality in South Asian countries by utilizing panel data from 2000–2017. The current study applied heterogeneous panel estimation techniques to examine the overlooked time-invariant heterogeneity and cross-dependency across countries.

The outcomes revealed that increased health expenditure and population growth were associated with an increased maternal mortality ratio. Health expenditure is a vital matter that can govern policy decisions at the national and international levels. Nevertheless, as indicated above, merely increasing health expenditure is insufficient; access, price, and consistency may influence the extent to which they are used, as well as, most importantly, their accessibility by deprived women. Thus, it is essential to go beyond simply expanding. Though increasing governments’ health care spending is essential for reducing mortality and enhancing health outcomes, the results for the sample countries indicate that governments should support the accountability and transparency of the amounts allocated for maternal health care services. In the nonexistence of transparent financial support, the possibility of achieving the SDG in South Asian countries will be difficult. This evidence should support officials in the health sector in countries with rising rates of maternal mortality to lower the mortality rate, either directly by increasing access to better health amenities for underprivileged women or indirectly by increasing the financial capability of officials to allow sufficient funds to the maternal health sector in general and programs for the prevention of mortality in particular [86]. The government should allocate a larger portion of the health budget for maternal health. Health services should accompany a suitable allocation of funds for women’s health at the community level. Women from resource-poor settings who are in utmost need of health advice and at the highest peril of death have limited health facilities. They can better avail these facilities if such services accessible adjacent to their home town, preferably in their neighborhoods. What is required is the placement of a number of health workers at the community level who are armed with proper training for maternal health care. This is possibly one of the best methods for taking advantage of resources. Additionally, our outcomes also focus on the significance of economic growth, and access to sanitation and clean fuel technologies in lowering maternal mortality. The results indicate that the governments of South Asian countries should improve the infrastructure and increase access to water and sanitation to end preventable deaths of mothers. The prevention of maternal deaths requires extensive socioeconomic modifications beyond the boundaries of the health care system alone, and these aspects can make childbirth and pregnancy safer naturally.

The findings of this study area new addition to the literature concerning health expenditure and maternal mortality in similar nations of South Asia. The findings show that each region has its challenges for health, and how sensibly expenditure on health is directed is the crux of the matter. Another area of action is improving the quality of health care facilities and competent people. This encompasses more than guaranteeing equipment availability; it also requires making the health services more publicly liable. The prevailing healthcare system adds to the rising maternal mortality due to meager and incompetent arrangements or meager administrative competencies. It also reflects the priorities established by an elitist system in which the powerless and poor do not count.

This study has also certain notable limitations. For example, the analysis only added a few control variables and ignored other potential confounding variables, such as the literacy rate, the physician to population ratio, and many other aspects of health. The reason for this was the high rate of missing data for these variables, which resulted in the exclusion of these variables from the model. Moreover, it is also likely that not all of the indicators of the World Bank databank are recounted each year. Thus the World Bank data might not remain consistent with the annually reported data of the Ministry of Health of each country. Further, the World Development data only give the data at the national level and, in this case, may not be helpful for showing the situation at the individual and community levels. Moreover, the present study utilized data from an South Asian panel, so the outcome may not be generalized to other developing countries. Regardless of these limitations, it is expected that the current study will provide impactful and meaningful results for policy makers to devise strategies, keeping in view the fundamental South Asian aspects warranted to reduce maternal mortality.

## Figures and Tables

**Table 1:** Descriptive Statistics.

	Maternal Mortality	Health Expenditure	Economic Growth	Population	Clean Fuel Technologies	Basic Sanitation
Mean	312.4028	105.0910	24.18627	1.834289	32.16650	53.63868
Median	236.5000	40.11737	24.24980	1.562703	26.56000	48.65390
Maximum	1450.000	946.8112	28.60913	4.668361	94.56000	99.37300
Minimum	36.00000	8.362380	20.31048	−0.266960	7.240000	15.12425
Std. Dev.	302.9920	184.0360	2.209372	1.067289	20.24544	23.83500
Skewness	1.957352	2.931312	0.159694	0.850876	1.503814	0.484218
Kurtosis	6.467059	10.90132	2.181728	3.393813	4.979517	2.010786
Jarque–Bera	164.0724	580.8078	4.629465	18.30628	77.24570	11.49848
Probability	0.000000	0.000000	0.098793	0.000106	0.000000	0.003185

Source: Authors’ estimations.

**Table 2:** Results of the stationarity analysis.

Variable	Im, Pesaran, and Shin			
	I (0)		I (1)	
	Intercept	Intercept with Trend	Intercept	Intercept with Trend
Maternal Mortality	−3.44394 ***	1.28452	−3.37734 ***	−6.50037 ***
Health Expenditure	6.53287	−1.45462 *	−5.33655 ***	−5.31860 ***
Economic Growth	1.32175	−20.9478 ***	−33.4276 ***	−30.2320 ***
Population	−3.65753 ***	−7.00708 ***	−9.68864 ***	−12.4179 ***
Basic Sanitation	5.17881	1.69497	−4.75597 ***	−17.7165 ***
Clean Technologies	3.39259	1.21140	−4.34171 ***	−9.37003 ***

Note: ***, * show significance at the 1% and 10% levels, respectively.

**Table 3:** Cross-sectional dependence and CIPS unit root test results.

Variables	CD Test	*p*-Value	CIPS Test	
			Level	First Difference
Maternal Mortality	22.01677	0.000	−2.606 ***	−4.253 ***
Health Expenditure	21.45441	0.000	−0.909	−3.654 ***
Economic Growth	16.55560	0.000	−2.653 ***	−4.603 ***
Population	5.181234	0.000	−3.334 ***	−1.659 ***
Basic Sanitation	22.39049	0.000	−0.451	−2.028 *
Clean Technologies	21.89715	0.000	−0.269	−3.935 ***

Note: ***, * signifies significance at the 1% and 10% levels, respectively.

**Table 4:** Results of Pedroni co-integration (Engle-Granger based) in the South Asian panel.

MMR = f (HE+ LNGDP + POP + BS+ CT)		
Estimates	Stats.	Prob.
Panel v-statistics	−0.4468	0.673
Panel rho-statistics	2.04059	0.979
Panel PP-statistics	−11.393	0.000
Panel ADF-statistics	−6.6653	0.000
Alternative hypothesis: individual AR coefficient		
Group rho-statistic	2.97338	0.999
Group PP-statistic	−14.826	0.000
Group ADF-statistic	−3.5834	0.002

Note: The panel cointegration null hypothesis is no cointegration.

**Table 5:** Panel regression results.

Fixed Effect					Random Effect			
Variables	Coefficient	Std. Error	T-Stat	*p*-Value	Coefficient	Std. Error	T-Stat	*p*-Value
Health Expenditure	0.4347	0.1183	3.67	0.000	0.4284	0.1190	3.6	0.000
Economic Growth	58.853	13.533	4.35	0.000	44.911	12.554	3.58	0.000
Population	71.887	12.736	5.64	0.000	72.051	12.923	5.58	0.000
Basic Sanitation	−4.415	1.0606	−4.16	0.000	−4.258	1.0396	−4.1	0.000
Clean Fuel Technologies	−8.577	1.4936	−5.74	0.000	−8.273	1.4802	−5.59	0.000
Constant	−775.0	320.65	−2.42	0.017	−456.6	308.21	−1.48	0.138
R^2^	0.2783				R^2^	0.3399		
Prob > F	0.0000				Prob > chi-sq	0.0000		
Hausman test Prob > chi2	0.0000							

Source: Estimations by the authors.

**Table 6:** FMOLS and DOLS long-run results.

	FMOLS			DOLS		
Variables	Coefficient	Std. Error	T-Stat	Coefficient	Std. Error	T-Stat
Health Expenditure	1.9592 ***	0.1910	10.255	0.1675 **	0.0803	2.0813
Economic Growth	−94.264 ***	26.727	−3.526	−18.22	13.392	−1.360
Population	40.640 ***	7.3916	5.4981	10.61 **	4.9330	2.1513
Basic Sanitation	−7.2306 **	3.2193	−2.245	−1.960 **	0.8554	−2.291
Clean Fuel Technologies	−11.581 ***	1.4931	−7.756	−5.691 ***	1.0351	−5.498

Note: ***, ** indicates significance at the 1% and 5% levels, respectively.

## Data Availability

The data will be available on request.
